# NGS Gene Panel Analysis Revealed Novel Mutations in Patients with Rare Congenital Diarrheal Disorders

**DOI:** 10.3390/diagnostics11020262

**Published:** 2021-02-08

**Authors:** Maria Valeria Esposito, Marika Comegna, Gustavo Cernera, Monica Gelzo, Lorella Paparo, Roberto Berni Canani, Giuseppe Castaldo

**Affiliations:** 1 CEINGE-Biotecnologie Avanzate, 80145 Naples, Italy; espositomari@ceinge.unina.it (M.V.E.); comegna@ceinge.unina.it (M.C.); cernera@ceinge.unina.it (G.C.); gelzo@ceinge.unina.it (M.G.); berni@unina.it (R.B.C.); 2 Dipartimento di Medicina Molecolare e Biotecnologie Mediche, Università di Napoli Federico II, 80131 Naples, Italy; 3 Dipartimento di Scienze Mediche Traslazionali, Università di Napoli Federico II, 80131 Naples, Italy; lorella.paparo@unina.it

**Keywords:** NGS, congenital diarrhea disorders, genes panel

## Abstract

Congenital diarrheal disorders (CDDs) are early-onset enteropathies generally inherited as autosomal recessive traits. Most patients with CDDs require rapid diagnosis as they need immediate and specific therapy to avoid a poor prognosis, but their clinical picture is often overlapping with a myriad of nongenetic diarrheal diseases. We developed a next-generation sequencing (NGS) panel for the analysis of 92 CDD-related genes, by which we analyzed patients suspect for CDD, among which were (i) three patients with sucrose-isomaltase deficiency; (ii) four patients with microvillous inclusion disease; (iii) five patients with congenital tufting enteropathy; (iv) eight patients with glucose-galactose malabsorption; (v) five patients with congenital chloride diarrhea. In all cases, we identified the mutations in the disease-gene, among which were several novel mutations for which we defined pathogenicity using a combination of bioinformatic tools. Although CDDs are rare, all together, they have an incidence of about 1%. Considering that the clinical picture of these disorders is often confusing, a CDD-related multigene NGS panel contributes to unequivocal and rapid diagnosis, which also reduces the need for invasive procedures.

## 1. Introduction

Congenital diarrheal disorders (CDDs) are a heterogeneous group of rare enteropathies characterized by early-onset, generally monogenic and inherited as an autosomal recessive trait [1]. In many CDD forms, diarrhea appears as the main symptom, while, in other cases, it appears as a corollary of a more complex, systemic, and multiorgan syndrome [2,3]. Most patients with CDDs require a rapid diagnosis since they need immediate and specific therapy to avoid a poor outcome [2,3]. The diagnostic approach may be complex because of the large number of conditions, even nongenetic conditions, in differential diagnosis [1,2,4]. Some CDDs appear with a specific clinical picture, and there are tests that allow us to quickly achieve the diagnosis, while, in other forms, the symptoms may overlap, and no tests other than genetic analysis are available [5]. 

For many CDDs, the disease-gene is known; therefore, molecular analysis can provide a rapid and specific diagnostic contribution [2]. Furthermore, mutation analysis helps predict the severity of the course through genotype–phenotype correlations or, in some cases, to guide the therapeutic choice [6,7]. In addition, molecular analysis allows us to carry-out genetic counseling to the family, perform carrier analysis, and offer prenatal diagnosis to high-risk couples [8].

In our laboratory, over the last 10 years, we have developed a flowchart for the diagnosis of CDDs [1,2,3,4], and we carried out molecular analyses for a dozen different CDDs. Given that most forms of CDD are clinically indistinguishable (thus requiring the contextual analysis of multiple genes), we developed a next-generation sequencing (NGS) panel for the analysis of all genes related to CDDs that we now describe, together with the results of molecular analysis of several patients with rare CDD-bearing novel genotypes. 

## 2. Materials and Methods

### 2.1. Samples Collection

We studied 25 patients suspected to have CDDs, who showed severe, chronic diarrhea starting from the first months of life (in most cases, since the first days), associated with different combinations of vomiting, dehydration, failure to thrive, abdominal distention, and acid-base balance alterations, mostly metabolic acidosis (Supplementary Table S1). After an evaluation aimed to exclude nongenetic causes of diarrhea [4] and to diagnose a CDD [5], such patients were referred to our laboratory for molecular analysis of one or more disease-genes related to CDDs (Table 1). All enrolled subjects (legal guardians for minors) underwent pretest counseling during which they were informed about the significance of molecular analysis, provided information about their personal and familial history, and gave written informed consent for the anonymous use of their clinical data. For all enrolled subjects, we recorded the data on their personal and familial history and their clinical conditions. 

A blood EDTA sample was collected from each subject. Genomic DNA (gDNA) was isolated from peripheral blood using the Nucleon BACC3 Genomic DNA Extraction Kit (GE Healthcare, Life Sciences, Chicago, IL, USA) or with the robotic workstation MagnaPure (Roche, Basel, Switzerland) for fully automated purification of nucleic acids, according to the manufacturer’s instructions. The quality of DNA samples was assessed by the TapeStation system (Agilent Technologies, Santa Clara, CA, USA); only gDNA samples with a DNA integrity number (DIN) >6 were considered suitable for NGS analysis. DNA quantity was evaluated through the NanoDrop 2000c spectrophotometer (Thermo Fisher Scientific, Waltham, MA, USA) and by using Qubit dsDNA BR and HS assays kits (Life Technologies, Carlsbad, CA, USA). 

### 2.2. NGS Custom Panel Design and Panel Content

To achieve the greatest diagnostic sensitivity and specificity, we selected 92 CDD genes, reported in Supplementary Table S2. These genes included several classes of CDD [1,2]: (i) genes involved in defects in epithelial nutrient and electrolyte transport; (ii) defects in epithelial enzymes and metabolism; (iii) defects in epithelial trafficking and polarity; (iv) enteroendocrine cell dysfunction; (v) immune dysregulation-associated enteropathy; (vi) related syndromes and chronic pancreatitis. For each gene, we analyzed the coding regions, 50 bp in each of the intronic boundaries, the promoter, and the 3′UTR for a total target size of about 1 Mb. 

However, some regions in 3′UTRs and promoters can consist of repeating regions in which the coverage can become lower and/or the variant filtering tools can exclude some variants because they are considered of poor quality. This could cause the loss of detection of some variants in these genomic regions.

### 2.3. NGS Library Preparation and Sequencing

Patient analysis was performed using the abovementioned NGS panel. The custom design of our probes was realized using the web-based SureDesign application (https://earray.chem.agilent.com/suredesign accessed on 12 July 2020). A total of 50 ng of gDNA was processed through the SureSelect^QXT^ Target Enrichment system (Agilent Technologies, Santa Clara, CA, USA) for Illumina multiplexed sequencing. Briefly, gDNA was enzymatically fragmented and adaptor-tagged to obtain a pool of fragments that were amplified by PCR reaction. Then, the prepared DNA library amplicons were hybridized to the capture custom library, made up of our 92 genes, and purified by streptavidin-coated magnetic beads. The captured, targeted-enriched DNA library was amplified by PCR reaction by using dual index primers, which allowed us to univocally barcode each sample. Finally, SureSelect-enriched dual-indexed NGS samples were pooled together for multiplexed sequencing. Sequencing reactions were carried out on the MiSeq instrument (Illumina, San Diego, CA, USA) using a PE 150 × 2 flow cell, running 16 samples for each sequencing run to obtain an average coverage of about 200× (>95% of the gene’s target nucleotides are covered at >100 reads, with mapping quality score (MQ > 30) reads); 96% of the analyzable target regions were covered by at least 50×.

### 2.4. NGS Data Analysis

The Alissa Align & Call v1.0.2.10 tool (Agilent Technologies, Santa Clara, CA, USA), using the genome build hg38 as a reference, was used to perform alignments, variant calling, and quality filtering. The median QV bases used in variant calling was 39, with an average read length of 141 bp.

Variant filtering and interpretation were done using Alissa Interpret v5.2.6 CE IVD software (Agilent Technologies, Santa Clara, CA, USA), using GRCh38.p2 and annotation sources like 1000 Genomes (Phase 3 release v5, 10 September 2014, including GRCh38 data), ClinVar (NCBI ClinVar October 2019), DGV (Database of Genomic Variants, version 15 May 2016), ESP6500 (variants in the ESP6500SI-V2 dataset of the exome sequencing project, annotated with SeattleSeqAnnotation137), ExAC (ExAC release 1.0—including GRCh38 from lift over data), OMIM (OMIM, version 25 October 2019), dbNSFP (dbNSFP v3.0b2: Database of functional predictions for nonsynonymous SNPs), dbSNP (dbSNP build 151), and gnomAD (gnomAD release 2.0.2).

### 2.5. Variant’s Pathogenicity Predictions

Bioinformatics predictions of the variant’s effects were performed using the SIFT (http://sift.jcvi.org/ accessed on 31 March 2020) and PolyPhen-2 (http://genetics.bwh.harvard.edu/pph2/ accessed on 31 March 2020) tools. Further predictions were assessed with the Mutation Taster tool (http://www.mutationtaster.org accessed on 31 March 2020) [9] and other tools included on the VarSome website (https://varsome.com/variant/hg38 accessed on 31 March 2020) [10,11]. All software were used with their default parameter. All pathogenic mutations and variants of unknown significance that had clinical relevance were confirmed with standard Sanger sequencing. 

Finally, to define the pathogenic role of the variants identified in our patients, we used the following approach: (i) we verified whether the variant had been previously identified in patients bearing the disease under study; (ii) we used the abovementioned prediction tools; (iii) for novel missense variants not annotated into databases, we searched for the variant in 200 alleles derived from normal subjects; (iv) in patients that resulted homozygous for a variant, in order to exclude a large deletion in the proband, we analyzed both the parents to verify that both were heterozygous. Variants classification was performed following American College of Medical Genetics and Genomics (ACMG) guidelines [12].

## 3. Results

### 3.1. Sucrase-Isomaltase (SI) Deficiency

Table 2 shows the results of the molecular analysis performed on three patients affected by SI deficiency (OMIM #222900). Case 1 is male and is compound heterozygous for the c.5234T>G and c.4474C>T SI gene mutations. Of these two mutations, the first causes the change of phenylalanine with arginine (p.Phe1745Cys) and has previously been described in a patient with SI deficiency [13]. The second is a novel variant that causes the formation of a premature stop codon (p.Arg1492Ter), and prediction analysis confirmed its pathogenic role (Supplementary Table S3). Case 2 is female, conceived by consanguineous parents, and is homozygous for the c.3218G>A mutation. This mutation causes the substitution of glycine with aspartate at position 1073 (p.Gly1073Asp). Both parents were heterozygous for the proband mutation. The possible pathogenic effect of such mutation, already described in patients with SI deficiency [13], was confirmed by bioinformatics tools (Supplementary Table S3). Finally, Case 3 is male and was referred for specific suspicion of SI deficiency. Molecular analysis revealed c.2074C>T and no other mutations in the SI gene. The pathogenic role of the mutation was defined by bioinformatic predictions (Supplementary Table S3).

### 3.2. Microvillus Inclusion Disease (MVID)

We studied four patients referred for suspected MVID (OMIM #251850; Table 3). The first two patients, both males, respectively homozygous for the c.505A>G mutation (Case 1) and compound heterozygous for the c.1376A>G and c.2700delG mutations (Case 2), had been previously described by our group [14]. Case 3 is female and molecular analysis of the myosin (MYO)5B gene revealed c.577C>A and the c.656G>A variants, both previously reported in MVID patients [14] and both predicted to be pathogenic (Supplementary Table S3). Case 4 is female, born to consanguineous parents. She is homozygous for the c.413A>G variant in the MYO5B gene. Both the parents are heterozygous for the variant. Bioinformatic tools confirmed the pathogenic role of the novel mutation (Supplementary Table S3).

### 3.3. Congenital Tufting Enteropathy (CTE)

We studied 5 patients affected by CTE (OMIM # 613217; Table 4). Case 1, male, conceived by consanguineous parents, was homozygous for the known c.757G>A epithelial cell adhesion molecule (EPCAM) gene mutation [15]. Both the parents were heterozygous for the proband mutation. Bioinformatic tools predicted the variant as pathogenic (Supplementary Table S3). The variant, not annotated so far into databases, was absent in 200 alleles from control subjects. Case 2 was also born from consanguineous parents and homozygous for the novel c.712G>T variant. Again, both the parents were heterozygous for the c.712G>T variant. Such a variant causes the formation of a premature stop-codon (p.Glu238Ter) and is predicted to be pathogenic. Case 3 was compound heterozygous for the abovementioned c.712G>T mutation and the novel c.551-1G>T. The latter was predicted to be pathogenic since it impairs the splicing process. Case 4 was compound heterozygous for the novel c.649G>T EPCAM variant, which was predicted to be pathogenic because of the formation of a premature stop-codon (p.Glu217Ter), and for the c.758A>G, variant, predicted to be pathogenic by bioinformatic tools. Such mutation involves the same codon of the known c.757G>A pathogenic variant [15]. The c.758A>G variant was absent in 200 alleles from control subjects. Finally, in Case 5, only the EPCAM c.556-14A>G known mutation [15] was revealed. In this patient, the diagnosis of CTE was confirmed by enzymatic analysis of duodenal biopsy samples.

### 3.4. Glucose-Galactose Malabsorption (GGM)

We studied six patients suspected to have GGM (OMIM #606824; Table 5). Case 1, conceived by consanguineous parents, was homozygous for the solute carrier (SLC)5A1 gene c.799C>T mutation. Both the parents were heterozygous for the proband mutation. Such a mutation, previously described in GGM patients [16], is pathogenic since it causes a premature stop-codon (p.Arg267Ter). Similarly, Case 2, again born to consanguineous parents, was homozygous for a known [17] stop mutation, predicted as pathogenic (i.e., p.Arg63Ter), and present in both parents. Case 3 was compound heterozygous for two novel variants, i.e., c.637G>C and c.1028T>C. Both were predicted as pathogenic. The first, i.e., c.637G>C, not annotated into databases, was absent in 200 alleles from healthy subjects. Case 4, born of consanguineous parents, was homozygous for the novel p.Val370del mutation. Both the parents were heterozygous for the mutation, thus excluding a large deletion in the proband. The mutation was classified as pathogenic by bioinformatic tools and was absent in 200 alleles from healthy subjects. Case 5 is compound heterozygous for the known c.1845C>G mutation [18] and for the novel c.418C>T mutation, both predicted as pathogenic (Supplementary Table S3) by bioinformatic tools. Nevertheless, according the ACMG guidelines, the latter variants described in Case 5 should be considered variants of uncertain significance, although they appear to be causative of the disease phenotype. Finally, Case 6 is compound heterozygous for two novel variants, i.e., c.866G>A, which causes a premature stop-codon (i.e., p.Trp289Ter), and c.1573C>A, both predicted to be pathogenic. The second variant, not annotated into databases, was absent in 200 alleles from healthy subjects. 

In addition, we studied two subjects suspect to have GGM. The first, Case 7, carried 3 missense mutations in cis, forming a complex allele with no other mutations in trans. These variants are c.152A>G (p.Asn51Ser), c.1231G>A (p.Ala411Thr), and c.1845C>G (p.His615Gln). In particular, the p.Asn51Ser variant affects amino acid residues that are highly conserved across the SLC5A1 orthologs; hence, this mutation may be responsible for impaired sugar transport, while p.Ala411Thr and p.His615Gln are probably benign polymorphisms, according to Mutation Taster in-silico predictions. The other patient, Case 8, had the three mutations in cis and c.2T>A (p.Met1Lys) in trans. This variant has not been reported in reference databases and, according to in silico predictions, can be classified as pathogenic (i.e., Polyphen, SIFT, Mutation Taster, and other tools all agree on its pathogenicity). In both patients, the diagnosis of GGM was confirmed by histology of small bowel and hydrogen breath tests, suggesting that the first patient (Case 7) would have a second unidentified mutation within the noncoding regions of the gene, while in the other (Case 8), the complex allele and the c.2T>A mutations were responsible for the disease. However, the study of 200 alleles from healthy subjects revealed two cases heterozygous for c.152A>G (p.Asn51Ser), c.1231G>A (p.Ala411Thr), and c.1845C>G (p.His615Gln) mutations in cis. Furthermore, a sibling of Case 8, asymptomatic and negative to the hydrogen test, had the same genotype as the affected sibling, i.e., was compound heterozygous for c.152A>G (p.Asn51Ser), c.1231G>A (p.Ala411Thr), and c.1845C>G (p.His615Gln) complex alleles and c.2T>A in trans.

### 3.5. Congenital Chloride Diarrhea (CCD)

We recently described 12 novel solute carrier (SLC)26A3 mutations in 17 cases of CCDs (OMIM #214700) [19]. In the present study, we describe 5 other cases (Table 6). Case 1 is compound heterozygous for two novel gene variants, i.e., c.1484A>C and c.1181G>T, both predicted as pathogenic (Supplementary Table S3). The first variant has not been annotated into databases; we excluded its presence in 200 alleles from healthy subjects. Case 2, conceived by consanguineous parents, is homozygous for the novel c.614delT variant that causes the formation of a premature stop-codon (i.e., p.Leu205ArgfsTer28). The variant was predicted as pathogenic by bioinformatics tools (Supplementary Table S3). Both the parents were heterozygous for the proband mutation. Case 3 is compound heterozygous for the same single-nucleotide deletion, c.614delT, and for a known duplication, i.e., c.2024_2026dup, previously described [19]. Case 4 is compound heterozygous for the known18 c.358G>A mutation and for the novel c.1522T>C variant, predicted as pathogenic. Such a mutation is not annotated, and we excluded its presence in 200 alleles from healthy subjects. Finally, Case 5 is compound heterozygous for two known stop-codon mutations, c.559G>T and c.1735C>T (i.e., p.Gly187Ter and p.Arg579Ter).

## 4. Discussion

The NGS panel that we have developed facilitates the diagnostic workup of CDDs, providing an unequivocal diagnosis in patients that often require to be rapidly managed with specific therapies to avoid a poor outcome [3]. However, the analysis of large gene panels frequently reveals a number of variants of uncertain significance (VUS) and novel variants not reported into reference databases. Currently, the classification of these VUS as pathogenic involves the guidelines indicated by the American College of Medical Genetics, which are based on several criteria, including family studies, type of mutation, protein residue affected by the variants, genetic association studies, posterior-probabilities analysis, and functional studies [12]. Although these criteria are widely accepted and used, the classification of VUS is still a challenge due to their low frequency, the lack of family information, and the difficulty of performing functional studies using ex-vivo cell models [20], enteroids, or stem cells [21] in a routine context. In the present study, we assessed the pathogenic role of the novel variants by using the ACMG classification first, and then, by the main prediction tools currently used, as described in the methods section. Furthermore, a great help in the diagnosis of CDDs came from the continuous interaction between physicians and molecular biologists who have discussed each case potentially affected by CDDs and from the use of first-level diagnostic approaches to restrict the clinical suspects [5], an approach that has been developed by our team over the last 10 years. Finally, the present study permitted us to define the genotype of all 25 patients and to define the pathogenic role of two novel mutations responsible for SI deficiency, four novel mutations responsible for MVID, three for CTE, six for GGM, and four for CCDs, all very rare diseases for which a few dozen mutations are known so far [14,15,19,22].

Molecular analysis also helps to reduce the number of invasive approaches that could be required for infants or neonates. In fact, the deficiency of SI diagnosis is based on enzymatic analysis of biopsy samples from intestinal villi [23]. Similarly, the diagnosis of MVID is based on histology that evidences microvillus inclusion in up to 10% of intestinal villi of affected patients. The analysis is sometimes challenging [14,24], and the alterations may be absent in atypical forms of MVID [25]. CTE is due to villous atrophy with crypt hyperplasia and focal crowding of surface enterocytes that resemble tufts, evidenced by histology of intestinal samples, total or partial villus atrophy, and crypt hyperplasia in the absence of inflammation, with the typical focal epithelial tufts that permit the differential diagnosis between MVID and CTE [15]. The diagnosis of GGM is based on a combination of tests that includes stool sugar analysis, hydrogen exhalation, and small bowel histology [26]. In patients with CCDs, molecular analysis may help define the therapeutic strategy. In fact, we demonstrate that butyrate limits the severity of diarrhea [6], modulating intestine inflammation [27] and enhancing the expression of the SLC26A3 protein, particularly in patients with some mutations [7]. Thus, mutation analysis is crucial for the diagnosis of such disease but also to predict patient responsiveness to oral butyrate therapy.

However, despite the fact that NGS analysis includes all known genes related to CDDs, in some patients, only one mutation was identified. For example, in one of the three patients with SI deficiency, we identified only the c.2074C>T heterozygous mutation within the *SI* gene, but the diagnosis was confirmed by enzymatic analysis on duodenal biopsy samples [23]. Similarly, in a patient with MVID, the analysis revealed only the *EPCAM* c.556-14A>G known mutation [15]. In this patient, the diagnosis of CTE was confirmed by the enzymatic analysis on duodenal biopsy samples. It is possible that in these patients, a second, undetected mutation would be intronic or lie within the promoter [28] or within the 3′ untranslated region (UTR) of the gene [29], which are not covered by NGS. Otherwise, the patient with SI deficiency could bear to the novel potential entity of subjects that are affected by the disease but would result as heterozygous for SI gene mutations [13]. Moreover, these not-detected variants may also be located in other genes, which have not been strictly associated with CDDs to date. For instance, they may be located in other genes involved in the same pathways as the known associated ones. In this regard, to better achieve differential diagnosis in our routinely diagnostic procedures, we recently designed a new CDD-related panel of 112 genes. These include the 92 genes described herein and other genes predicted to be involved in these diseases; moreover, we also selected not only genes closely associated with CCDs but also those related to diseases that cause similar clinical features. The inclusion of genes predicted to be involved in CDD-related diseases may add intriguing insights into CCD pathogenesis and may help to achieve a more precise diagnosis.

An interesting point is the discordance of the genotype–phenotype analysis. Among the patients referred for GGM deficiency, two siblings had the same genotype, i.e., the complex allele c.152A>G, c.1231G>A, and c.1845C>G in trans, with the c.2T>A GGM mutation. Between the two siblings, only one was finally affected by GGM deficiency, while the other was not affected, despite the fact that the same genotype that included either c.152A>G or c.2T>A mutation was predicted as pathogenic. This case adds to the well-known variability of the impact of complex alleles [30,31] and to the different expression of the disease in sib-pairs that have the same genotype [31], which we recently demonstrated in patients with cystic fibrosis. 

To conclude: although most CDDs are rare, all together, they have an incidence of about 1% in the general population; the disease-genes is known in most cases, and unequivocal and rapid diagnosis is mandatory in most patients with CDDs in order to immediately start the specific therapy. 

Targeted gene panel analysis has pros and cons. The analysis of only “a few genes” compared to the thousands of genes that make up the exome is useful principally when clinical suspicion is strong and related to a limited number of diseases. Gene panel analysis allows us to reach a faster diagnosis, which, in some cases, can be decisive for the patient’s life. In contrast, exome analysis allows us to obtain a huge amount of data that can be used in the future and reanalyzed in the light of new scientific discoveries, but it requires great bioinformatics knowledge and longer analysis times. For these reasons, the exome analysis should be preferred when clinical suspicion is unclear. This is not feasible with diseases for which a very rapid therapeutic and clinical intervention is required. Nowadays, the cost of a gene panel and of an exome is absolutely comparable; the choice between one method and the other may depend on various parameters, such as the laboratory organization, the close collaboration between clinicians, geneticists, and molecular biologists, and the urgency with which a response is required.

In this context, a multigene NGS panel to simultaneously analyze all disease-genes responsible for CDDs is contributory in a reference laboratory for molecular diagnostics; however, adequate bioinformatics expertise and functional approaches to define the effect of novel mutations are ancillary and indispensable in the clinical context. Finally, a multidisciplinary approach between physicians and molecular geneticists to each patient suspected to have CDD is mandatory to offer a proper diagnostic service in this field.

## Figures and Tables

**Table 1:** Congenital diarrheal disorders and corresponding disease-genes analyzed in the present study.

Disease	Acronym	OMIM#	Gene	OMIM#
Congenital sucrase-isomaltase deficiency	CSID	222900	*Sucrase-Isomaltase*	609845
Microvillus inclusion disease	MVID	251850	*MYO5B*	606540
Congenital tufting enteropathy	CTE	613217	*EPCAM*	185535
Glucose-galactose malabsorption	GGM	606824	*SLC5A1*	182380
Congenital chloride diarrhea	CCD	214700	*SLC26A3*	126650

**Table 2:** Molecular analysis of patients with congenital sucrase-isomaltase deficiency included in the present study.

Case	Gender	Mutation			
		IUPAC Protein	HGVS Protein	HGVS Nucleotide	Reference Number NCBI
1	Male	F1745C	p.Phe1745Cys	c.5234T>G	79717168
		R1492X ^†^	p.Arg1492Ter	c.4474C>T	747584061
2	Female	G1073D	p.Gly1073Asp	c.3218G>A	121912616
		G1073D			
3	Male	R692C ^†^	p.Arg692Cys	c.2074C>T	371618948

^†^ Novel mutation.

**Table 3:** Molecular analysis of patients with microvillus inclusion disease included in the present study.

Case	Gender	Mutation			
		IUPAC Protein	HGVS Protein	HGVS Nucleotide	Reference Number NCBI
1	Male	K169E ^†^	p.Lys169Glu	c.505A>G	not reported
		K169E ^†^			
2	Male	N456S ^†^	p.Asn456Ser	c.1376A>G	1207737174
		R900S ^†^	p.Arg900SerfsTer4	c.2700delG	not reported
3	Female	S186X	p.Ser186Ter	c.557C>A	753977426
		R219H	p.Arg219His	c.656G>A	1053713532
4	Female	H138R ^†^	p.His138Arg	c.413A>G	1229761410
		H138R ^†^			

^†^ Novel mutation.

**Table 4:** Molecular analysis of patients with congenital tufting enteropathy included in the present study.

Case	Gender	Mutation			
		IUPAC Protein	HGVS Protein	HGVS Nucleotide	Reference Number NCBI
1	Male	D253N	p.Asp253Asn	c.757G>A	not reported
		D253N			
2	Male	E238X ^†^	p.Glu238Ter	c.712G>T	not reported
		E238X ^†^			
3	Female			c.551-1G>C^†^	not reported
		E238X ^†^	p.Glu238Ter	c.712G>T	not reported
4	Male	E217X ^†^	p.Glu217Ter	c.649G>T	not reported
		D253G ^†^	p.Asp253Gly	c.758A>G	not reported
5	Male		p.Tyr186PhefsTer6	c.556-14A>G	376155665

^†^ Novel mutation.

**Table 5:** Molecular analysis of patients with glucose-galactose malabsorption included in the present study.

Case	Gender	Mutation			
		IUPAC Protein	HGVS Protein	HGVS Nucleotide	Reference Number NCBI
1	Male	R267X	p.Arg267Ter	c.799C>T	779502629
		R267X			
2	Male	R63X	p.Arg63Ter	c.187C>T	202166715
		R63X			
3	Male	V213L ^†^	p.Val213Leu	c.637G>C	not reported
		I343T ^†^	p.Ile343Thr	c.1028T>C	774741107
4	Female	V370del ^†^	p.Val371del	c.1107_1109delAGT	not reported
		V370del ^†^			
5	Male	R140W ^†^	p.Arg140Trp	c.418C>T	748242943
		H615Q	p.His615Gln	c.1845C>G	33954001
6	Female	T289X ^†^	p.Trp289Ter	c.866G>A	755654536
		H525N ^†^	p.His525Asn	c.1573C>A	not reported
7	Male	N51S	p.Asn51Ser	c.152A>G	17683011
		A411T	p.Ala411Thr	c.1231G>A	17683430
		H615Q	p.His615Gln	c.1845C>G	33954001
8	Male	N51S	p.Asn51Ser	c.152A>G	17683011
		A411T	p.Ala411Thr	c.1231G>A	17683430
		H615Q	p.His615Gln	c.1845C>G	33954001
		M1K	p.Met1Lys	c.2T>A	not reported

^†^ Novel mutation.

**Table 6:** Molecular analysis of patients with congenital chloride diarrhea (CCD) included in the present study.

Case	Gender	Mutation			
		IUPAC Protein	HGVS Protein	HGVS Nucleotide	Reference Number NCBI
1	Male	Q495P ^†^	p.Gln495Pro	c.1484A>C	not reported
		S394I ^†^	p.Ser394Ile	c.1181G>T	1228273365
2	Male	L205RfsX28 ^†^	p.Leu205ArgfsTer28	c.614delT	1264217866
		L205RfsX28 ^†^			
3	Male	I675dup	p.Ile675dup	c.2024_2026dup	121913031
		L205RfsX28 ^†^	p.Leu205ArgfsTer28	c.614delT	1264217866
4	Female	C508R ^†^	p.Cys508Arg	c.1522T>C	not reported
		G120S	p.Gly120Ser	c.358G>A	386833479
5	Male	G187X	p.Gly187Ter	c.559G>T	121913032
		R579X	p.Arg579Ter	c.1735C>T	1171640656

^†^ Novel mutation.

## Data Availability

The authors confirm that the data supporting the findings of this study are available within the article and/or its supplementary material or can be made available upon reasonable request.

## Supplementary Materials

The following are available online at https://www.mdpi.com/2075-4418/11/2/262/s1. Supplementary Table S1 lists the clinical data of patients reported in this study. Supplementary Table S2 shows the list of genes included in our customized 92-gene panel. Supplementary Table S3 reports the database annotations and pathogenicity predictions of the variants reported in this study.
