# Supplementary material for: NGS Gene Panel Analysis Revealed Novel Mutations in Patients with Rare Congenital Diarrheal Disorders
Source: Diagnostics (Basel). 2021 Feb 8;11(2):262. doi: 10.3390/diagnostics11020262 (PMC7915612; doi:10.3390/diagnostics11020262)
Supplement: Supplementary file 1 [file diagnostics-11-00262-s001.zip › diagnostics-1100279-supplementary/Supplementary tables/New Supplemental Table 2.docx]

**Supplementary Table 2:** List of genes included in our customized 92-genes panel.

**Gene Transcript OMIM Associated phenotypes Inheritance**

**Epithelial nutrient and electrolyte transport**

SLC10A2 NM_000452.2 601295 Primary bile acid malabsorption AD

SLC26A3 NM_000111.2 126650 Congenital chloride diarrhea AR

SLC2A2 NM_000340.1 138160 Fanconi-Bickel syndrome AR/AD

SLC39A4 NM_017767.2 607059 Acrodermatitis enteropathica AR

SLC5A1 NM_000343.3 182380 Glucose-galactose malabsorption AR

SLC7A7 NM_001126106.2 603593 Lysinuric protein intolerance AR

SLC9A3 NM_004174.3 182307 Congenital secretory sodium diarrhea AR

GUCY2C NM_004963.3 601330 Guanylate cyclase 2C deficiency AD/AR

CFTR NM_000492.3 602421 Cystic Fibrosis AR

PERCC1 NM_001365310.2 618656 Malabsorptive diarrhea of infancy (DIAR 11) AR

WNT2B NM_024494.3 601968 Neonatal onset chronic diarrhea (DIAR 9) AR

**Epithelial enzymes and metabolism**

LCT NM_002299.2 603202 Lactase deficiency AR

SI NM_001041.3 609845 Sucrase-isomaltase deficiency AR

TMPRSS15 NM_002772.2 606635 Enteropeptidase deficiency AR

DGAT1 NM_012079.5 604900 Congenital protein-losing enteropathy AR

PLVAP NM_031310.2 607647 Congenital protein-losing enteropathy AR

MTTP NM_000253.3 157147 Abetalipoproteinemia AR

APOB NM_000384.2 107730 Familial hypercholesterolemia AR

NPC1L1 NM_013389.2 608010 LDLCQ7 AR

SAR1B NM_001033503.2 607690 Chylomicron retention disease AR

MGAM NM_001365693.1 154360 Maltase-Glucoamylase deficiency AR

MPI NM_001330372.1 154550 Congenital disorder of glycosylation type Ib AR

MVK NM_000431.2 251170 Mevalonic aciduria AD/AR

TCN2 NM_001184726.1 613441 Transcobalamin deficiency AR

PNLIP NM_000936.3 246600 Pancreatic lipase deficiency AR

ANGPTL3 NM_014495.4 604774 Hypobetalipoproteinemia AR

**Epithelial trafficking and polarity**

MYO5 B NM_001080467.2 606540 Microvillus inclusion disease AR

STX3 NM_004177.5 600876 Microvillus inclusion disease AR

EPCAM NM_002354.2 185535 Tufting enteropathy AD/AR

SPINT2 NM_021102.3 605124 Sodium congenital diarrhea AR

TTC37 NM_014639.3 614589 Syndromic diarrhea AR

TTC7A NM_001288951.1 609332 Gastrointestinal defects and immunodeficiency AR

**Enteroendocrine cell dysfunction**

NEUROG3 NM_020999.3 604882 Enteric anendocrinosis – AR

PCSK1 NM_000439.4 162150 Obesity with impaired prohormone processing AR

PCSK9 NM_174936.3 607786 Familial hypercholesterolemia, 3 AD

LIPA NM_000235.3 613497 Cholesterol ester storage disease AR

**Chronic Pancreatitis**

CTRC NM_007272.2 601405 Hereditary pancreatitis AD

PRSS1 NM_002769.4 276000 Hereditary pancreatitis AD/AR

PRSS2 NM_001303414.1 601564 Hereditary pancreatitis AD

SPINK1 NM_001379610.1 167790 Hereditary pancreatitis AD

**Immune dysregulation-associated enteropathy and related syndromes**

ADA NM_000022.2 608958 Adenosine deaminase deficiency AR

ADAM17 NM_003183.6 603639 Neonatal inflammatory skin and bowel disease AR

AIRE NM_000383.2 607358 APECED syndrome AR

ALPI NM_001631.4 171740 Inflammatory bowel disease AR

ANKZF1 NM_018089.2 617541 Infantile-onset inflammatory bowel disease AR

AP1S1 NM_001283.4 603531 MEDNIK syndrome AR

CARD11 NM_001324281.1 607210 Immunodeficiency 11B with atopic dermatitis AD/AR

CCBE1 NM_133459.4 612753 Lymphangiectasia-lymphedema syndrome 1 AR

CD3D NM_000732.4 186790 Immunodeficiency 19 AR

CD3E NM_000733.3 186830 T-B severe combined immunodeficiency AR

CLMP NM_024769.3 611693 Congenital short bowel syndrome AR/XL

CYBB NM_000397.4 300481 X-linked mycobacteriosis XL

CYP27A1 NM_000784.3 606530 Cerebrotendinous xanthomatosis AR

DCLRE1C NM_001289079.1 605988 Omenn syndrome AR

DNAJC21 NM_194283.3 617048 Bone marrow failure syndrome 3 AR

DUOX2 NM_001363711.2 606759 Thyroid dyshormonogenesis 6 AR

EFL1 NM_001322845.1 617538 Shwachman-Diamond syndrome 2 AR

ELANE NM_001972.4 130130 Severe congenital neutropenia 1 AD

FLNA NM_001110556.2 300017 Congenital short bowel syndrome AR/XL

FOXP3 NM_014009.4 300292 IPEX XL

IKBKG NM_001099857.5 300248 Immunodeficiency 33 XL

IL10 NM_000572.3 124092 Graft vs host disease AD

IL10RA NM_001558.3 146933 Inflammatory bowel disease AR

IL10RB NM_000628.3 123889 Inflammatory bowel disease AR

IL12RB1 NM_001290024.1 601604 Primary biliary cholangitis AR

IL21 NM_021803.4 605384 IL21-related infantile inflammatory bowel disease AR

IL2RA NM_000417.2 147730 Immunodeficiency due to CD25 deficiency AR

IL2RG NM_000206.3 308380 Immunodeficiency due to gamma chain deficiency XL

IL7R NM_002185.2 146661 IL-7R alpha deficiency AR

JAK3 NM_000215.3 600173 SCID AR

LRBA NM_001364905.1 606453 Common variable immunodeficiency 8 with autoimmunity AR

NCF1 NM_000265.5 608512 Chronic granulomatous disease AR/XL

NCF2 NM_000433.3 608515 Chronic granulomatous disease AR

NCF4 NM_013416.3 601488 Chronic granulomatous disease AR/XL

NHEJ1 NM_024782.2 611290 SCID with microcephaly AR

PNP NM_000270.3 164050 Immunodeficiency due to PNP deficiency AR

PTPRC NM_002838.4 151460 T-B severe combined immunodeficiency AR

RAG1 NM_000448.2 179615 Omenn syndrome AR

RAG2 NM_001243786.1 179616 Omenn syndrome AR

SBDS NM_016038.4 607444 Shwachman-Diamond syndrome AR

SERPINA1 NM_001127701.1 107400 AAT deficiency AR

SKIV2L NM_006929.4 600478 Trichohepatoenteric syndrome 2 AR

SRP54 NM_003136.3 604857 Shwachman-Diamond syndrome 2 AR

STAT1 NM_007315.3 600555 Autoimmune enteropathy and endocrinopathy AD

STAT5B NM_012448.4 604260 Laron syndrome with immunodeficiency -

TGFB1 NM_000660.6 190180 Inflammatory bowel disease AR

TNFAIP3 NM_001270508.1 191163 Familial autoinflammatory syndrome AD

UBR1 NM_174916.2 605981 Johanson-Blizzard syndrome AR

WAS NM_000377.3 300392 Severe congenital, X-linked Neutropenia, XR

XIAP NM_001167.3 300079 Lymphoproliferative syndrome XR

ZAP70 NM_001079.4 176947 Immunodeficiency 48 AR
