# Supplementary material for: NGS Gene Panel Analysis Revealed Novel Mutations in Patients with Rare Congenital Diarrheal Disorders
Source: Diagnostics (Basel). 2021 Feb 8;11(2):262. doi: 10.3390/diagnostics11020262 (PMC7915612; doi:10.3390/diagnostics11020262)
Supplement: Supplementary file 1 [file diagnostics-11-00262-s001.zip › diagnostics-1100279-supplementary/Supplementary tables/New Supplemental Table 3.docx]

Supplementary Table 3. Database annotations and pathogenicity predictions of variants reported in this study. ^†^Novel mutation. P is pathogenic, D is damaging, DC is disease causing, LP is likely pathogenic, CI is conflicting interpretations, US is uncertain significance, T is tolerated, B is benign, LB is likely benign, NR is not reported. ACMG classification refers to American College of Medical Genetics and Genomics; Richards S. et al., Genet Med 2015. The mutation nomenclature is referred to HGVS.

**Gene Mutation *In silico* pathogenicity predictions**

**Protein Nucleotide NCBI ClinVar ACMG Mutation Taster SIFT**

SI p.Phe1745Cys c.5234T>G rs79717168 CI LP DC D

p.Arg1492Ter^†^ c.4474C>T rs747584061 NR P DC D

SI p.Gly1073Asp c.3218G>A rs121912616 LP LP DC D p.Gly1073Asp

SI p.Arg692Cys^†^ c.2074C>T rs371618948 NR LP DC D

MYO5B p.Lys169Glu^†^ c.505A>G not reported NR US DC D

p.Lys169Glu^†^

MYO5B p.Asn456Ser^†^ c.1376A>G rs1207737174 NR P DC D

p.Arg900SerfsTer4^†^ c.2700delG not reported NR P DC D

MYO5B p.Ser186Ter c.557C>A rs753977426 NR P DC D

p.Arg219His c.656G>A rs1053713532 P LP DC D

MYO5B p.His138Arg^†^ c.413A>G rs1229761410 NR US DC D

p.His138Arg^†^

EPCAM p.Asp253Asn c.757G>A not reported NR US DC D

p.Asp253Asn

EPCAM p.Glu238Ter^†^ c.712G>T not reported NR P DC D

p.Glu238Ter^†^

EPCAM c.551-1G>C^†^ not reported NR P DC D

p.Glu238Ter^†^ c.712G>T not reported NR P DC D

EPCAM p.Glu217Ter^†^ c.649G>T not reported NR P DC D

p.Asp253Gly^†^ c.758A>G not reported NR US DC D

EPCAM p.Tyr186Phefs*6 c.556-14A>G rs376155665 P P DC D

SLC5A1 p.Arg267Ter c.799C>T rs779502629 P P DC D

p.Arg267Ter

SLC5A1 p.Arg63Ter c.187C>T rs202166715 NR P DC D

p.Arg63Ter

SLC5A1 p.Val213Leu^†^ c.637G>C not reported NR US P T

p.Ile343Thr^†^ c.1028T>C rs774741107 US US DC D

SLC5A1 p.Val371del^†^ c.1107_1109delAGT not reported NR LP DC D

p.Val371del^†^

SLC5A1 p.Arg140Trp^†^ c.418C>T rs748242943 NR US DC D

p.His615Gln c.1845C>G rs33954001 LB B P T

SLC5A1 p.Trp289Ter^†^ c.866G>A rs755654536 NR LP DC D

p.His525Asn^†^ c.1573C>A not reported NR LP DC D

SLC26A3 p.Gln495Pro^†^ c.1484A>C not reported NR LP DC D

p.Ser394Ile^†^ c.1181G>T rs1228273365 NR LP DC D

SLC26A3 p.Leu205ArgfsTer28^†^ c.614delT rs1264217866 NR LP DC D

p.Leu205ArgfsTer28^†^

SLC26A3 p.Ile675dup c.2024_2026dup rs121913031 P LP DC D

p.Leu205ArgfsTer28^†^ c.614delT rs1264217866 NR LP DC D

SLC26A3 p.Cys508Arg^†^ c.1522T>C not reported NR P DC D

p.Gly120Ser c.358G>A rs386833479 LP P DC D

SLC26A3 p.Gly187Ter c.559G>T rs121913032 P P DC D

p.Arg579Ter c.1735C>T rs1171640656 P P DC D
