# Supplementary material for: NGS Gene Panel Analysis Revealed Novel Mutations in Patients with Rare Congenital Diarrheal Disorders
Source: Diagnostics (Basel). 2021 Feb 8;11(2):262. doi: 10.3390/diagnostics11020262 (PMC7915612; doi:10.3390/diagnostics11020262)
Supplement: Supplementary file 1 [file diagnostics-11-00262-s001.zip › diagnostics-1100279-supplementary/Supplementary tables/NEW Supplementary Table 1.docx]

| **Patient** | **Gender** | **Disease** | **Age at diarrhea onset (months)** | **Main clinical features** | **Main laboratory features** |
| --- | --- | --- | --- | --- | --- |
| 1 | M | CSID | 8 | Abdominal pain, bloating, excess gas production, [diarrhea](http://www.nlm.nih.gov/medlineplus/ency/article/003126.htm) | Serum electrolytes imbalance |
| 2 | F | CSID | 9 | Stomach cramps, abdominal distention, [diarrhea](http://www.nlm.nih.gov/medlineplus/ency/article/003126.htm) | Serum electrolytes imbalance |
| 3 | M | CSID | 7 | Bloating, vomiting, [diarrhea](http://www.nlm.nih.gov/medlineplus/ency/article/003126.htm) | Serum electrolytes imbalance |
| 4 | M | MVID | 1 | Watery diarrhea, abdominal distension, dehydration, failure to thrive, vomiting | Serum electrolytes imbalance, metabolic acidosis |
| 5 | M | MVID | 1 | Watery diarrhea, dehydration, failure to thrive, abdominal distension | Serum electrolytes imbalance, metabolic acidosis |
| 6 | F | MVID | 1 | Watery diarrhea, vomiting, irritability, abdominal distension, dehydration, failure to thrive | Serum electrolytes imbalance, metabolic acidosis |
| 7 | F | MVID | 1 | Watery diarrhea, abdominal distension, dehydration, failure to thrive | Serum electrolytes imbalance, metabolic acidosis |
| 8 | M | CTE | 1 | Vomiting, watery diarrhea, abdominal distension, dehydration, failure to thrive | Serum electrolytes imbalance |
| 9 | M | CTE | 2 | Diarrhea, abdominal distension, dehydration, failure to thrive | Serum electrolytes imbalance, metabolic acidosis |
| 10 | F | CTE | 1 | Diarrhea, abdominal distension, dehydration, failure to thrive | Serum electrolytes imbalance, metabolic acidosis |
| 11 | M | CTE | 1 | Watery diarrhea, vomiting, abdominal distension, dehydration, failure to thrive | Serum electrolytes imbalance, metabolic acidosis |
| 12 | M | CTE | 2 | Watery diarrhea, abdominal distension, dehydration, failure to thrive | Serum electrolytes imbalance, metabolic acidosis |
| 13 | M | GGM | 1 | Diarrhea, abdominal distension, dehydration, failure to thrive | Serum electrolytes imbalance, metabolic acidosis |
| 14 | M | GGM | 2 | Watery diarrhea, abdominal distension, dehydration, vomiting, failure to thrive | Serum electrolytes imbalance, metabolic acidosis |
| 15 | M | GGM | 2 | Diarrhea, abdominal distension, dehydration, failure to thrive | Serum electrolytes imbalance, metabolic acidosis |
| 16 | F | GGM | 1 | Watery diarrhea, abdominal distension, dehydration, failure to thrive | Serum electrolytes imbalance, metabolic acidosis |
| 17 | M | GGM | 2 | Watery diarrhea, abdominal distension, dehydration, failure to thrive | Serum electrolytes imbalance, metabolic acidosis |
| 18 | M | GGM | 1 | Watery diarrhea, abdominal distension, dehydration, failure to thrive | Serum electrolytes imbalance, metabolic acidosis |
| 19 | M | GGM | 1 | Watery diarrhea, abdominal distension, dehydration, failure to thrive | Serum electrolytes imbalance, metabolic acidosis |
| 20 | M | GGM | 2 | Watery diarrhea, abdominal distension, dehydration, failure to thrive | Serum electrolytes imbalance, metabolic acidosis |
| 21 | M | CCD | 1 | Watery diarrhea, abdominal distension, dehydration, failure to thrive | Metabolic alkalosis, serum electrolytes imbalance, hyper-reninemia and aldosterolemia |
| 22 | M | CCD | 1 | Watery diarrhea, abdominal distension, dehydration, failure to thrive | Serum electrolytes imbalance, metabolic alkalosis, hyper-reninemia/ aldosterolemia |
| 23 | F | CCD | 1 | Watery diarrhea, abdominal distension, dehydration, failure to thrive | Metabolic alkalosis, serum electrolytes imbalance, hyper-reninemia/ aldosterolemia |
| 24 | M | CCD | 1 | Watery diarrhea, abdominal distension, dehydration, failure to thrive | Metabolic alkalosis, serum electrolytes imbalance, hyper-reninemia/ aldosterolemia |
| 25 | M | CCD | 1 | Watery diarrhea, abdominal distension, dehydration, failure to thrive | Metabolic alkalosis, serum electrolytes imbalance, hyper-reninemia/ aldosterolemia |

**Note:** CSID, Congenital Sucrase-Isomaltase Deficiency; MVID, Microvillus Inclusion Disease; CTE, Congenital Tufting Enteropathy; GGM, Glucose-Galactose Malabsorption; CCD, Congenital Chloride Diarrhea
